# Case Report: A Case of Caprine Arthritis Encephalitis in Dairy Goat Farms in South Korea

**DOI:** 10.3389/fvets.2021.773039

**Published:** 2021-12-07

**Authors:** Ga-In Son, Eui-Ju Hong, Hyun-Jin Shin

**Affiliations:** ^1^College of Veterinary Medicine at Chungnam National University, Daejeon, South Korea; ^2^Research Institute of Veterinary Medicine at Chungnam National University, Daejeon, South Korea

**Keywords:** case report, caprine arthritis encephalitis (CAE), outbreak, Korea, dairy goats

## Abstract

One Saanen dairy goat (*Capra aegagrus hircus*) farm in Korea reported that some goats showed clinical signs such as arthritis, paralysis, carpal joint swelling, and even death. We monitored clinical signs and pathological lesions. In the laboratory, we confirmed caprine arthritis encephalitis virus (CAEV) infection by polymerase chain reaction (PCR). We examined all the dairy goats on the farm and found that many of them were positive. In conclusion, CAEV infection was detected in the majority of the goats in this farm, and it induced severe clinical signs impacting productivity and causing important economic shortfalls. We need to regularly investigate all dairy goat farms, and, more importantly, inspection of the quarantine stage should be required before importation. Interestingly, we found all negative results in Korean native black goats (*Capra hircus linnaeus*).

## Introduction

Caprine arthritis encephalitis virus (CAEV) is a single-stranded, icosahedral RNA virus of the family *Retroviridae* and belongs to subgroup B of small ruminant lentiviruses (SRLVs). Goats infected with CAEV exhibit severe and chronic devastating disease characterized by significant economic loss. There are four major clinical forms/presentations: arthritis, encephalitis, mastitis, and interstitial pneumonia (1–4). Infection with CAEV is persistent and lifelong. After being first diagnosed in goats in 1974, it has been diagnosed worldwide (5–8). The arthritic form is the most common and is generally observed in individuals aged 6 months and older (9, 10). Although it is a widespread disease in most goat-farming countries, little information is available on the CAEV infection status of Korean goats. The major route of transmission is considered vertical transmission through colostrum or infected milk. After ingestion in milk, the virus crosses the small intestine and infects monocytes and macrophages (1, 11). There is horizontal transmission by direct contact between goats through prolonged contact between infected and healthy animals housed in the same location (12). Sexual transmission is also another suspected route of transmission, and some sources speculate that *in utero* transmission occurs; however, additional studies and confirmation are still required (7, 13). Latent infection is common to most lentiviruses, similar to CAEV, for which the infection remains latent until monocytes mature into macrophages (1, 14).

The aim of our study was to investigate the clinical and pathological findings of caprine arthritis encephalitis (CAE) in dairy goats in Korea. Although there have been many similar reports, as there are a very small number of dairy goats in Korea, CAE reports are very rare. We previously published only genetic sequence information of CAEV in Korea, and we report our findings of clinical and pathological results in this report.

## Case Description

**Figure 1:**
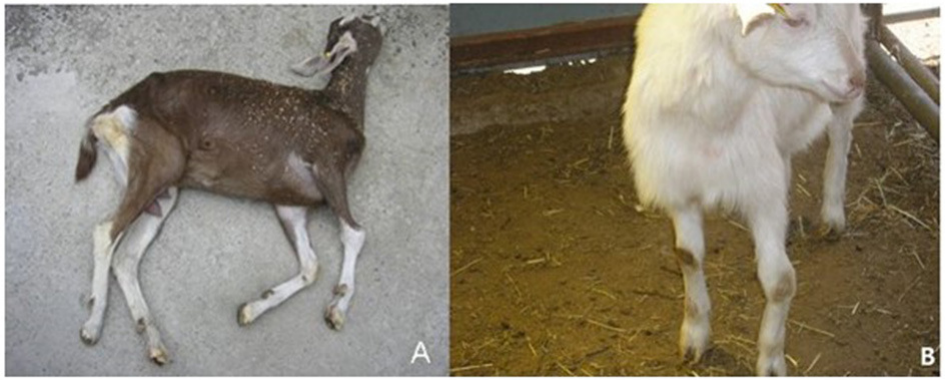
Clinical signs and lesions of CAEV-infected goats. The diseased goats showing clinical signs with encephalitis and arthritis of carpal joints. **(A)** Opisthotonus. **(B)** Swelling and abduction of carpal joints in adult goat.

Among the 111 goats tested, 56 goats were positive by PCR. In particular, one Saanen dairy goat farm in Korea reported that some goats showed clinical signs such as arthritis, paralysis, swelling of the carpal joints, and even death. With careful observation, we found that more than 60 adult goats showed similar clinical signs. The farm was isolated from villages in mountain areas. Clinical signs were found in 11 adults older than 6 months and 10 young animals between 6 months and 1 year old. As shown in Figures 1A,B, swelling of the carpal joints, stiffness, difficulty moving and standing, distortion of the leg, and cachexia were common in most goats suspected of being infected. Two adults older than 1 year had mastitis, arthritis, and a rapid drop in milk production. Farmers reported that the goats were found dead mostly within 1 month or after a maximum of 3–4 months after clinical signs started. In young kids (under 3–4 months old), the most common symptoms were pneumonia and nervous system signs. Perhaps because of nervous system signs, nervous system symptoms such as lameness, body paralysis, tremor, and torticollis were found (Figures 1A,B).

## Diagnosis and Confirmation Procedure

We collected leukocyte samples from all goats in the farm through the jugular vein and extracted total DNA from peripheral blood lymphocytes (PBLs) for CAEV detection by PCR. Peripheral blood mononuclear cells (PBMCs) were separated using the Ficoll gradient centrifugation method using Histopaque®1077-1 (Sigma Aldrich, USA). Briefly, 3 ml of leukocytes was placed onto Histopaque®1077. Centrifugation was performed at 400 × g for exactly 30 min at room temperature. After centrifugation, the interface was carefully transferred into a clean conical centrifuge tube. After washing, the cells were centrifuged at 250 × g for 10 min. The cell pellet was resuspended in 5 ml of isotonic phosphate-buffered saline solution or appropriate cell culture medium and mixed by gently pipetting in and out. The cell pellet was resuspended in 0.5 ml of isotonic phosphate-buffered saline solution. Total DNA was extracted using a G-DEX™ Genomic DNA Extraction Kit for blood (iNtRON, Korea) following the manufacturer's manual. DNA extraction from joint fluids was performed using a G-spin™ Genomic DNA Extraction Kit (iNtRON, Korea). Nested PCR was performed as described previously (15). Briefly, two sets of primers were used: the first PCR sense primer 5′-CAAGCAGCAGGAGGGAGAAGCTG-3′ and antisense primer 5′-TCCTACCCCCATAATTTGATCCAC-3′ were used to amplify a 296-bp PCR product. The conditions were 37 cycles of denaturation at 94°C for 2 min, annealing at 60°C for 10 s, and polymerization at 72°C for 30 s. The PCR product from the first reaction was used as a template for nested PCR. The primers used for nested PCR were the sense primer 5′-GTTCCAGCAACTGCAAACAGTAGCAATG-3′ and antisense primer 5′-ACCTTTCTGCTTCTTCATTTAATTTCCC-3′. They are positioned at nucleotides 997 and 1154 on the gag gene of the CAEV Cork strain. All PCR products were confirmed by sequencing analysis.

During clinical sign monitoring, some suspected goats showed clinical signs similar to CAE, such as arthritis and neurological dysfunction, and the infection was confirmed by nested PCR in the laboratory (7). Primer information and conditions for the nested PCR were the same as previously described (15).

We tested all 97 adult goats and found that 52 were positive for CAEV by PCR. We also randomly selected 14 kids and found that 4 were positive by PCR. Detailed information is provided in Table 1. Sequencing analysis showed ~96% similarity with CAEV (strain: Br/UFRGS-2/C767) and 91% similarity with ovine lentivirus (Visna-Maedi virus: strain: lt585). Detailed information was provided in our previous publication (15).

**Table 1:** PCR results of on the breed and sex data in the group of kids.

**Age**	**Breed**	**Sex**	**Number of CAEV positive goats/all tested goats**
Kids (up to 6 months)	Saanen	Both	4/14
1 year	Saanen	♀	15/28
2 years	Saanen	♀	17/34
3 years	Saanen	♀	20/35

**Figure 2:**
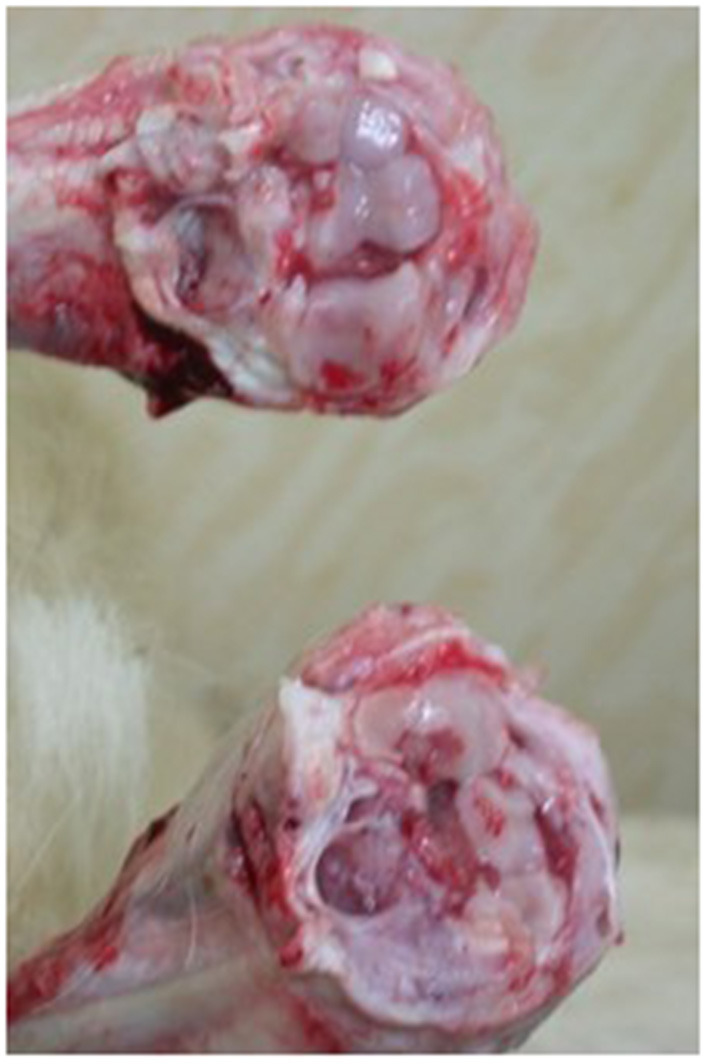
Pathological changes. Swelling and hardness of joint heads, articular cartilage ulceration, severely swollen mesenteric lymph nodes, and fibrinous arthritis were found.

**Figure 3:**
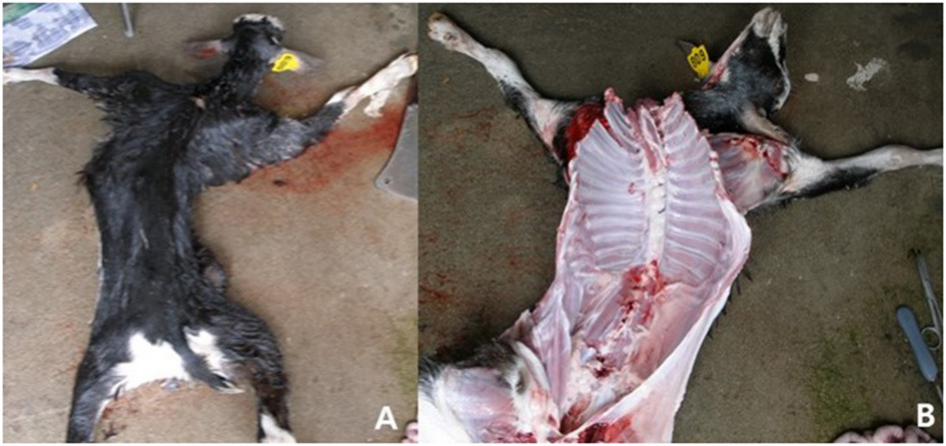
**(A,B)** Necropsy results. As the necropsy results, we found serious distortion of head and backbone and swelling of joints.

We necropsied one 8-month-old kid and observed severe lesions corresponding to CAEV infection. According to the necropsy results, pathological changes, such as swelling and hardness of joint heads, articular cartilage ulceration on the joint cavity, and severely swollen mesenteric lymph nodes, were found (Figure 2). We found serious distortion of the head and backbone and swelling of the carpal joints (Figure 3). We also collected tissue samples from the lung, liver, brain, kidney, or other organs and performed PCR. We found PCR positivity only in both the PBL and brain but negativity in all other tissues tested, such as the lung, liver, brain, kidney, spleen, and heart. We also investigated goats on two neighboring Saanen goat farms that had different ages, such as kids and 1- to 3-year-old adults. Interestingly, only the goats on the dairy goat farms were PCR positive, and PCR results were negative for six farms that had only Korean native black goats.

## Discussion

CAE is currently common to all dairy goat-producing countries worldwide, but there is still no effective vaccine or treatment (16). Studies from Japan have reported CAE detection by agar gel immunodiffusion test (AGID test) or enzyme-linked immunosorbent assay (ELISA), and all positive animals were slaughtered (10). Reports from Switzerland propose that all goats should have antibody testing for CAEV at least once per year (17). Antibody-positive goats are slaughtered, and farms are under special control and guidance. Only farms with three consecutive negative results are designated clean farms.

In our study, we found a 54% positive rate for CAEV by PCR from PBLs among adult animals in one farm tested. Additionally, we found a 29% positive rate among goat kids on the same farm. The tissue samples we tested were all negative except PBL and brain. The spleen was also negative, which is interesting because it is known to be rich in macrophages. We still need additional samples from other infected goats to confirm this result. Additionally, we are still unsure about how and when these goat kids developed CAEV infection. However, we have a clear notion that farmers isolated them before the kids took colostrum to prevent vertical transmission. It has been reported that even the blood of infected ewes is an important source for transmission, and vertical transmission has been proven (18). As goats are commonly imported from outside countries, they might become infected before they are exported. CAE is common in most dairy goats, but there have been no reports on Korean native black goats with clinical signs and pathological lesions except serological survey reports (19). Our report is the first report that Korean native black goats are resistant to CAEV infection. We did not find evidence of CAEV infections in farms with Korean native black goats located close to CAEV-infected dairy goat farms. This finding is indirect evidence suggesting that Korean native black goats may be resistant to CAEV infection. Additional investigations will be needed to prove this point.

## Conclusion

In conclusion, as CAEV infection outbreaks seem to spread rapidly to goats on a farm, they seem to have a serious economic impact. We need to regularly investigate all dairy goat farms and, more importantly, inspect the quarantine stage. Although we found all negative results in Korean native black goats on farms neighboring infected dairy goat farms, to confirm whether Korean black goats are resistant to CAE, survey studies using ELISAs might be required.

## Data Availability Statement

The original contributions presented in the study are included in the article/supplementary material, further inquiries can be directed to the corresponding author.

## Ethics Statement

Ethical review and approval was not required for the animal study because this is only field case not the animal experiments with infection. Written informed consent was obtained from the owners for the participation of their animals in this study.

## Author Contributions

HJS supervised this project, designed the experiments, and prepared the manuscript. GIS collected samples and performed the experiments. EJH provided technical support. All authors have read and approved the final manuscript.

## Funding

This work was supported by the Chungnam National University, South Korea.

## Conflict of Interest

The authors declare that the research was conducted in the absence of any commercial or financial relationships that could be construed as a potential conflict of interest.

## Publisher's Note

All claims expressed in this article are solely those of the authors and do not necessarily represent those of their affiliated organizations, or those of the publisher, the editors and the reviewers. Any product that may be evaluated in this article, or claim that may be made by its manufacturer, is not guaranteed or endorsed by the publisher.
